# Application of CD56, P63 and CK19 immunohistochemistry in the diagnosis of papillary carcinoma of the thyroid

**DOI:** 10.1186/1746-1596-3-5

**Published:** 2008-02-06

**Authors:** Dina El Demellawy, Ahmed Nasr, Salem Alowami

**Affiliations:** 1 University of Northern Ontario School of Medicine, Thunder Bay Regional Health Sciences Centre, Department of Pathology and Laboratory Medicine, Thunder Bay, Ontario, Canada; 2 University of Toronto, Department of Surgery, Toronto, Ontario, Canada; 3 McMaster University, Department of Pathology and Molecular Medicine, Hamilton, Ontario, Canada

## Abstract

Papillary carcinoma of the thyroid (PTC) is the commonest thyroid cancer. In the recent decades an obvious increase in the incidence of PTC has occurred. The pathological diagnosis of PTC is usually an easy diagnosis in the majority of cases. However since the introduction of follicular variant of PTC and the wide threshold range in interpretation of the clearly set pathological criteria for diagnosis of PTC, between pathologists including experts, the diagnosis in some cases became quite difficult. Unfortunately some cases are unjustifiably over-called as follicular variant of PTC as a result of the wide inter observable variability between pathologists, including thyroid pathologists.

Ancillary studies such as immmunohistochemistry may be helpful, but till now there is no 100% consistent marker(s), that distinct between PTC and other follicular thyroid lesions and tumors.

We assessed expression of antibodies against CD56, CK19, P63 and E-Cadherin in PTC and other follicular thyroid lesions and neoplasms. A total of 175 cases were studied. The neoplastic cases included 75 carcinomas (72 papillary, 2 follicular, 1 Hurthle cell) and 35 adenomas (32 follicular and 3 Hurthle cell). The non-neoplastic thyroids included 65 cases, (25 nodular hyperplasia, 5 thyrotoxic hyperplasia (Grave's disease), 19 lymphocytic thyroiditis and 6 Hashimoto's thyroiditis). All cases were evaluated by immunohistochemistry for the expression of the above mentioned markers. The markers' patterns and intensities of staining were scored. Positive expression of the markers equal or >10% of the follicular epithelium within the tumor or lesional cells was considered positive. An expression of <10% was considered to be negative.

Our results showed CD56 positive in all the lesions and tumors except for PTC in all cases (100%). CD56 was negative in all PTC cases (100%). CK 19 showed positive expression in PTC accounting for 85% of cases and in 26% of non PTC lesions/tumors. P63 showed selective focal positivity in PTC cases, in contrast to other non PTC lesions/tumors. P63 expression was in 70% of cases of PTC and was consistently absent in all the non PTC cases. E-Cadherin showed consistent non discriminatory expression in all cases included in the study.

We concluded that a panel consisted of CD56, CK19 and P63 is of value in distinction of PTC from other thyroid follicular lesion. P63 is a specific but less sensitive marker for PTC than CK19. CD56 is more specific and sensitive marker than CK19, however it is a negative rather than a positive marker for PTC. E-Cadherin is of no value in the diagnosis of thyroid follicular lesions/tumors. We recommend application of a panel composed of CK19, P63 and CD56 by a group of expert thyroid pathologists on a large series of follicular malignant thyroid neoplasms of uncertain malignant.

## Introduction

Papillary thyroid carcinoma (PTC) is the commonest thyroid cancer and through the recent decades a marked increase in its incidence has occurred. Such increase reflects true increase in incidence of PTC with a minor component of over diagnosis of PTC. It is clear that some cases do raise controversy as being PTC or non PTC. For example follicular adenoma and follicular variant of PTC, when some of the nuclear diagnostic criteria for PTC are occasionally present. Unfortunately such controversy exists between expert thyroid pathologists. Inter observer disagreements among pathologists are welldocumented [1]. Eight American and Japanese pathologists had only a 62% diagnosticagreement of 21 thyroid nodules [2]. It should be noted that these cases represent a minority of cases and commonly represented by follicular variant of PTC (as mentioned) or PTC arising in a setting of Hashimoto's thyroiditis. Although these cases are minority of cases, labeling patients with cancer and their over management is unacceptable, even though it protects the pathologists from facing an under diagnosed PTC with future metastasis. Despite that the diagnostic criteria for PTC have been established for more than 50 years, [3,4] it seems that its application, especially as regards quantization is still not fully established.

Up till today the gold standard for diagnosis of follicular thyroid lesions particularly PTC is histology. Some of the ancillary studies as immunohistochemistry and molecular techniques may be helpful, but none of them is conclusive. Hence the diagnosis of PTC in some cases still subjective with Inter observer variation between expert thyroid pathologists that varies between benignity and malignancy on the same case.

CD56 is a neural cell adhesion molecule; hence its expression may affect the migratory capability of tumor cells. Hence it is not surprising that loss of CD56 correlates with metastatic potentials and poor prognostic outcome in some malignancies [5,6].

P63, a p53-homologue nuclear transcription factor that is located on 3q27 and encodes six different isoforms, which harbor either trans-activating or negative dominant effects on p53 reporter genes. P63 tumor suppressing properties are disputable and mutations in this gene are rather rare in human malignancies [7,8]. It is consistently expressed in basal, squamous and myoepithelial cells such as in basal cells of the prostate acini and ducts, myoepithelial cells of the breast and squamous cell carcinoma [7-11].

Cytokeratin polypeptide 19 (CK19) is a type I intermediate filament protein an is the smallest known keratin and is remarkable in that, contrary to all other keratins, it does not have a designated partner for the formation of filaments, implying that regulation of its expression is different from other keratin-encoding genes [12]. Cytokeratin 19 concentrate at sarcomeres of striated muscle and copurify with the dystrophinglycoprotein complex, perhaps through the interaction of the cytokeratin with the actinbinding domain of dystrophin. In vitro studies showed that dystrophin binds directly and specifically to CK19 [13]. CK19 is synthesized in simple and stratified epithelia. E-cadherin is a molecule involved in adhesion between epithelial cells that seems to have a protective role in cancer, since its loss is associated with tumor progression and metastases formation in a series of different cancers [14].

We evaluated the diagnostic value of proteins expressions using antibodies against CD56, P63, CK19 and E-Cadherin in normal follicular thyroid epithelium, follicular thyroid lesions, and follicular thyroid neoplasms. Our aim was to study the applicability of difference in these markers' expressions that distinguish PTC, including the follicular variant from other follicular thyroid lesions and neoplasms, provided that the right morphological and clinical features are fulfilled.

## Methods

Thyroid gland lesions and tumors from 1999 to 2007 were searched through the Anatomic Pathology database and the electronic patients' charts at Thunder Bay Regional Health Sciences Center and Hamilton Health Sciences Center.

Demographic information, clinical data, tumor stage, treatment, and follow up were reviewed. Cases were reviewed by two pathologists (SA and DD). For the diagnosis of PTC, we followed the same histological criteria applied to the diagnosis of PTC as those proposed by Chan JKC [15], which are divided into major and minor features. The major features include: 1. Nuclei are ovoid rather than round; 2. Nuclei are crowded, often manifesting as lack of polarization in the cells that line a follicle; 3. Nuclei show a clear or pale chromatin pattern; 4. Psammoma bodies are found. If one of the 4 features is lacking, 4 or more of the following subsidiary features may occur: 1. Presence of abortive papillae; 2. Predominantly elongated or irregularly shaped follicles; 3. Dark-staining colloid; 4. Presence of rare nuclear pseudoinclusions; or 5. Multinucleated histiocytes in the lumens of follicles.

The cases diagnosed as Hürthle cell tumors/carcinomas were composed of greater than 75% oncocytic cells showing moderate to abundant eosinophilic granular cytoplasm as proposed in an earlier study [16]. Adenomas were defined as completely encapsulated follicular or Hürthle cell tumors with homogeneous architecture and morphology, lacking nuclear features of PTC and without capsular and vascular invasion [16].

As per the local surgical pathology protocols, submitted representative tissue sections from resections specimens were routinely processed and embedded in paraffin. In cases which show a grossly distinct nodule, the nodule is submitted in toto. Sections 2–3 μ were stained for immunohistochemistry with a standard avidin-biotin complex method using antibodies against CD56, P63, CK19 and E-Cadherin (table 1). Results of immunostains were assessed by two pathologists (DD and SA), and a consensus regarding controversial cases was reached at the double-headed microscope. Evaluation of the immunohistochemical staining was performed by light microscopy using a 10× objective lens with the selective use of a 20–40× objective lens for confirmation. A positive membranous expression with or without cytoplasmic taining in 10% or more of neoplastic cells qualified the case as "positive (+)" for CD56, E-Cadherin and CK19. Any nuclear P63 staining is accounted as positive expression of P63. Negative cases for P63 are that shows zero nuclear staining for P63 in the lesional/neoplastic cells. Immunohistochemistry was performed on 4 m thick sections using the labeled streptavidin-biotin peroxidase complex system (LSAB2) in a Dako Autostainer (Dako, Carpinteria, CA, USA).

**Table 1:** Properties of immunhistochemical antibodies used in the study

**Primary Antibody**	**Marker/Clone**	**Supplier**	**Mono (M) Poly (P)**	**Dilution**	**Antigen Retrieval**
CD56	Clone: 123C3	Zymed Invitrogen Corp. (USA)	M	1/100	pH 9.0
E-Cadherin	Clone: NCH-38	Dako USA	M	1/100	pH 9.0
Cytokeratin 19	Clone RCK108	Dako USA	M	1/50	Enzyme Proteinase K
P63	Clone: 4A4	Dako USA	M	1/100	pH 9.0

## Results

Out of the 203 cases of thyroid resections retrieved, a total of 185 cases were included in the study and all were stained for antibodies against CD56, P63, CK19 and ECadherin. A total of 18 cases were excluded as these represented medullary (10 cases) and anaplastic (4 cases) thyroid carcinomas, those classified as follicular neoplasm of uncertain malignant potential (3 cases) or caused controversy during review regarding diagnosis (1 case). The 185 cases included 175 cases of follicular lesions and neoplasms and 10 cases of normal thyroids from radical laryngectomies for laryngeal squamous cell carcinomas. The 175 follicular lesions and neoplasms included 75 carcinomas (72 papillary, 2 follicular, 1 Hurthle cell) and 35 adenomas (32 follicular and 3 Hurthle cell).

The PTC included 15 classic, 23 follicular, 27 micropapillary (24 follicular micropapillary and 3 classic micropapillary), 2 Hurthle cell (oxyphilic), one columnar cell, 2 encapsulated and 2 tall cell variants. None of the cases of PTC was diffuse sclerosing, cribriform, trabecular, muco-epidermoid, PTC with nodular fasciitis-like stroma or dedifferentiated PTC variant. Nine cases of PTC (5 from follicular variant, 1 from columnar variant and 3 from classic variant) showed metastatic cervical lymph nodes during presentation or follow up. In both instances metastasis was documented through pathological examination. Cases of PTC did not show another site of metastasis other than cervical lymph nodes.

The non-neoplastic thyroids included 65 cases, (25 nodular hyperplasia, 5 thyrotoxic hyperplasia (Grave's disease), 19 lymphocytic thyroiditis and 6 Hashimoto's thyroiditis). Immunohistochemical results.

**Figure 1:**
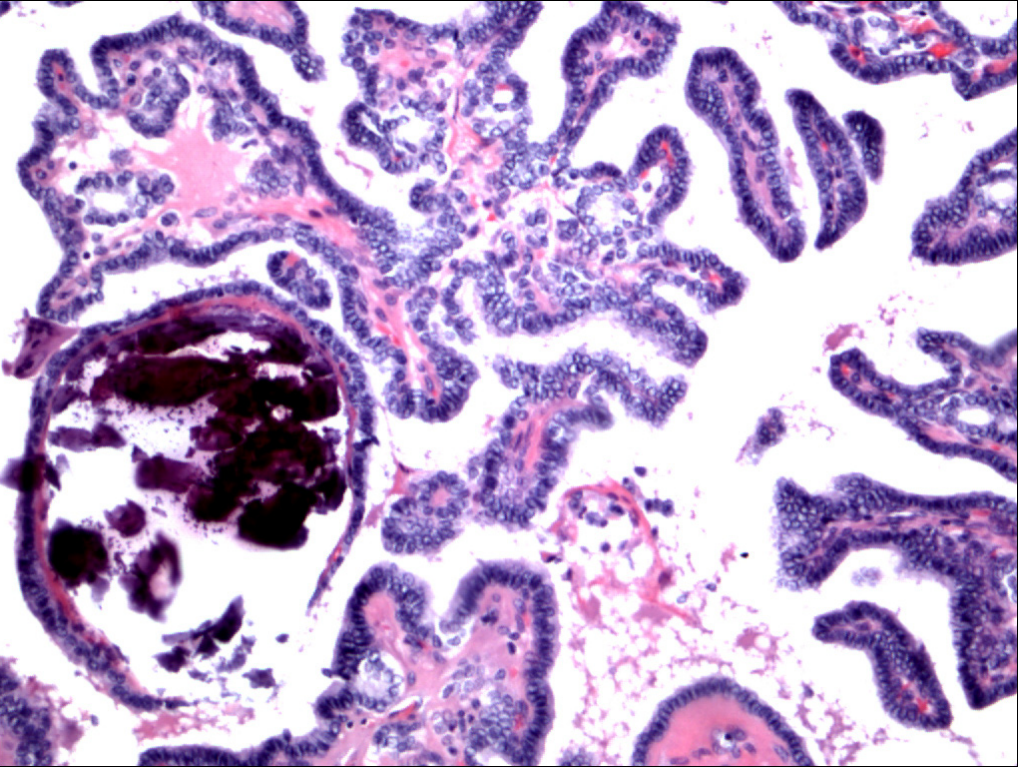
Classic papillary carcinoma H&E.

**Figure 2:**
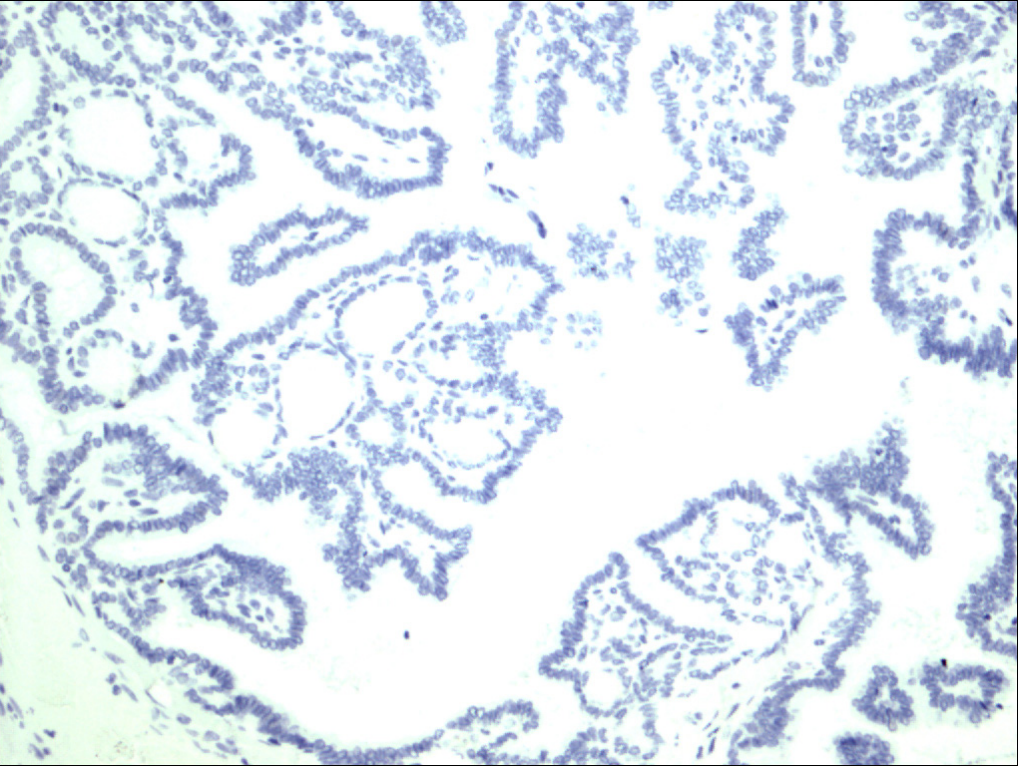
Classic papillary carcinoma CD56.

**Figure 3:**
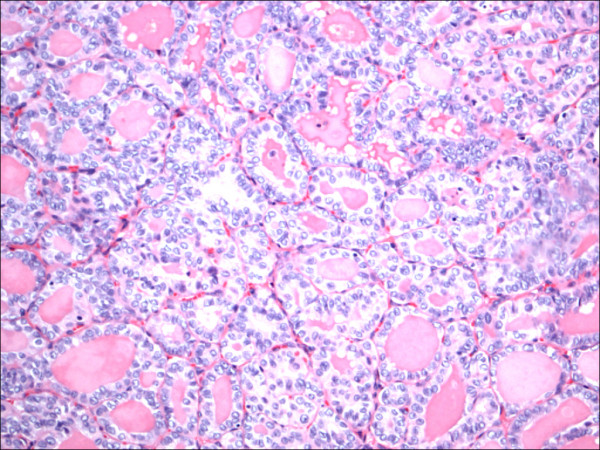
Follicular variant papillary carcinoma H&E.

**Figure 4:**
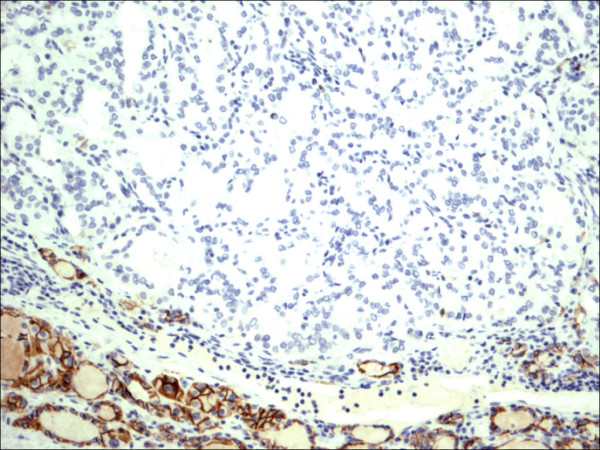
Follicular variant papillary carcinoma CD56.

**Figure 5:**
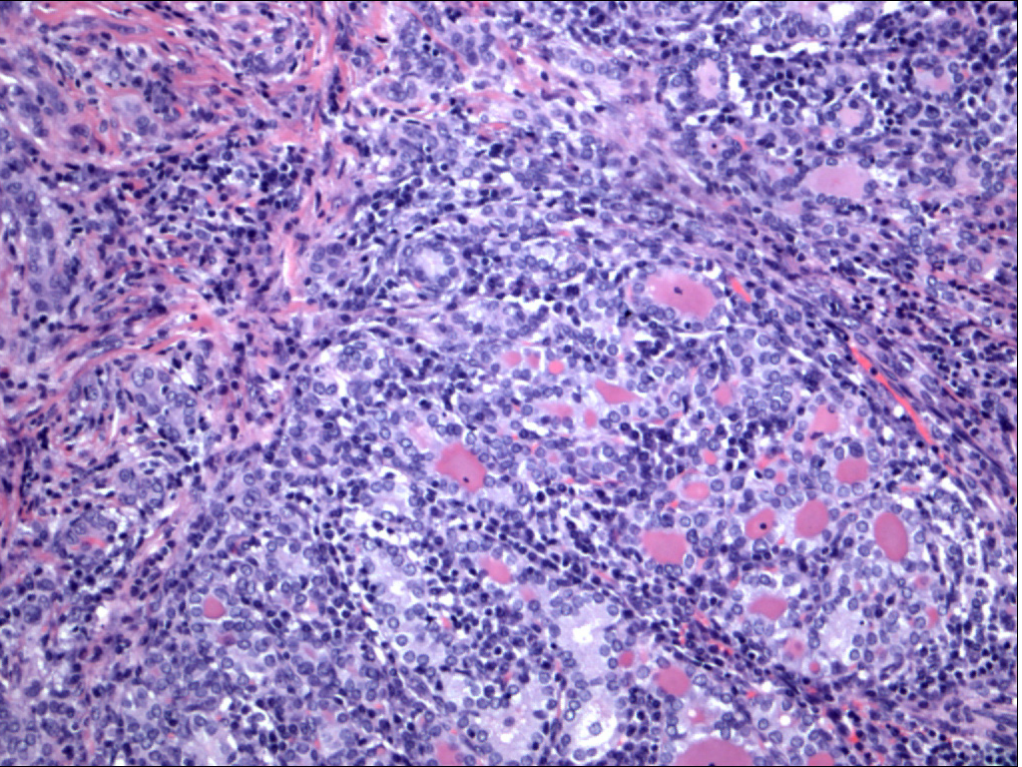
Papillary carcinoma in the setting of Hashimoto thyroiditis H&E.

**Figure 6:**
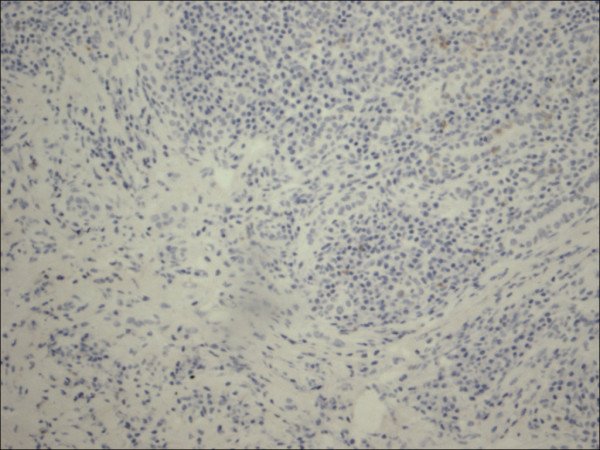
Papillary carcinoma in the setting of Hashimoto thyroiditis CD56.

The expression of CD56 proteins was diffuse and strong within all thyrocytes except for all cases of PTC (Figure 1, Figure 2, Figure 3, Figure 4, Figure 5 and Figure 6), including the encapsulated, follicular and microcarcinoma variants.

Out of the 175 cases included in the study, only the cases of PTC (including the encapsulated, follicular (Figure 3, Figure 4), and microcarcinoma variants and those occurring in the setting of Hashimoto's thyroiditis (Figure 5, Figure 6) showed absent CD56 expression. Such finding was consistent in all the cases and showed no difference with different tumor morphological variant or tumor stage. The metastatic deposits of PTC within the lymph nodes showed absent CD56 expression, as seen in their thyroid primaries. The surrounding non-lesional thyrocytes showed identical CD56 staining properties.

**Figure 7:**
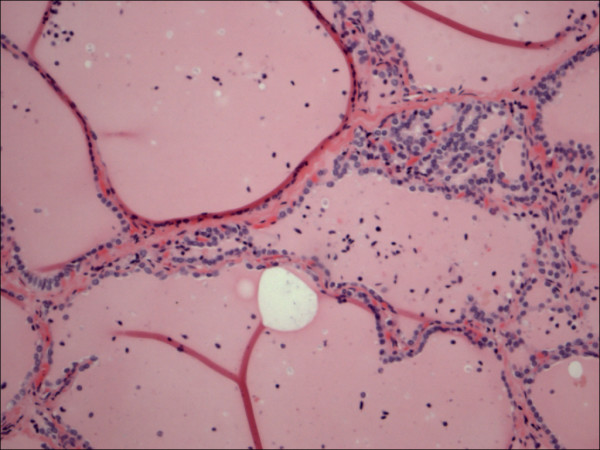
Thyrotoxicosis H&E.

**Figure 8:**
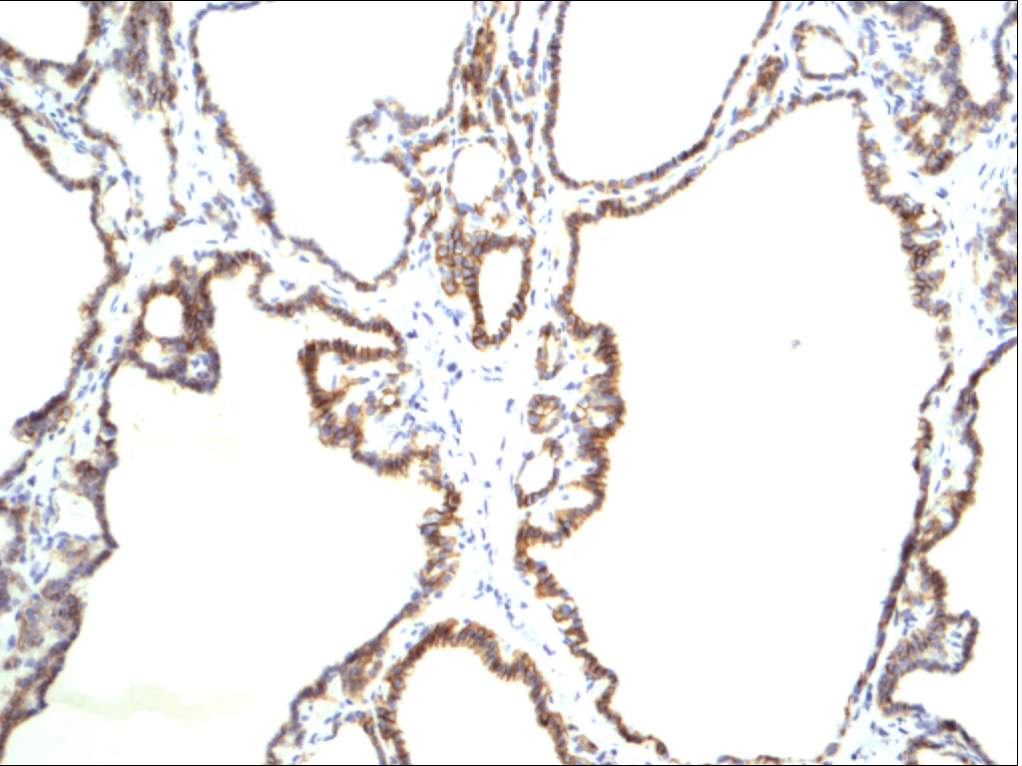
Thyrotoxicosis CD56.

**Figure 9:**
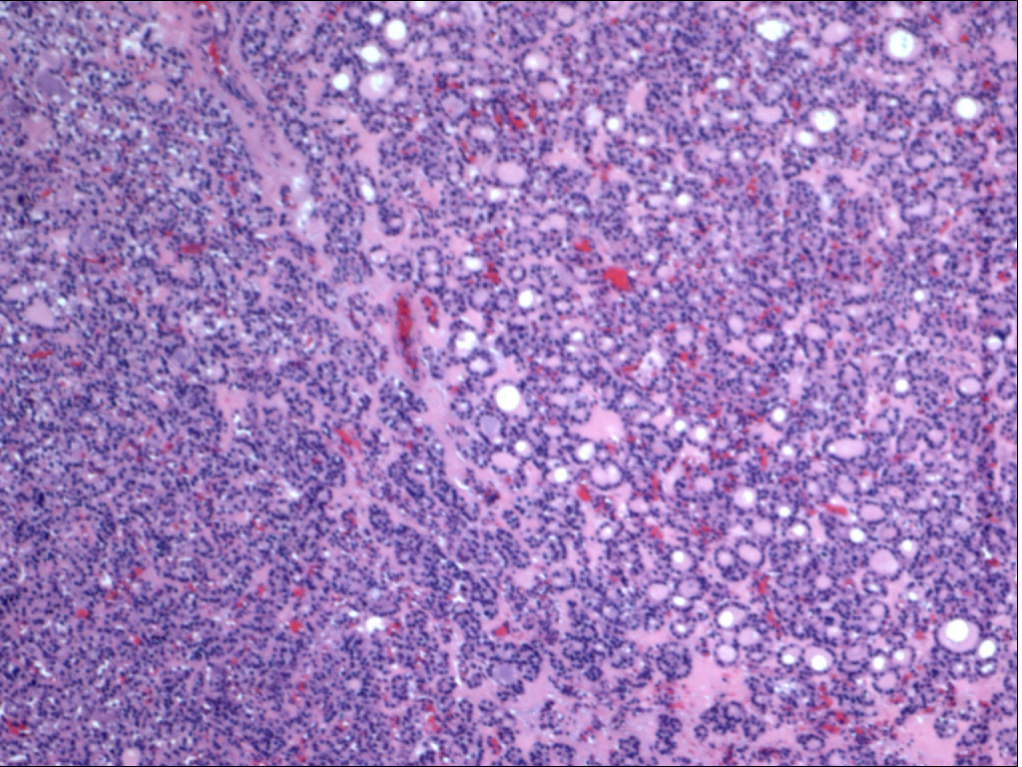
Follicular adenoma H&E.

**Figure 10:**
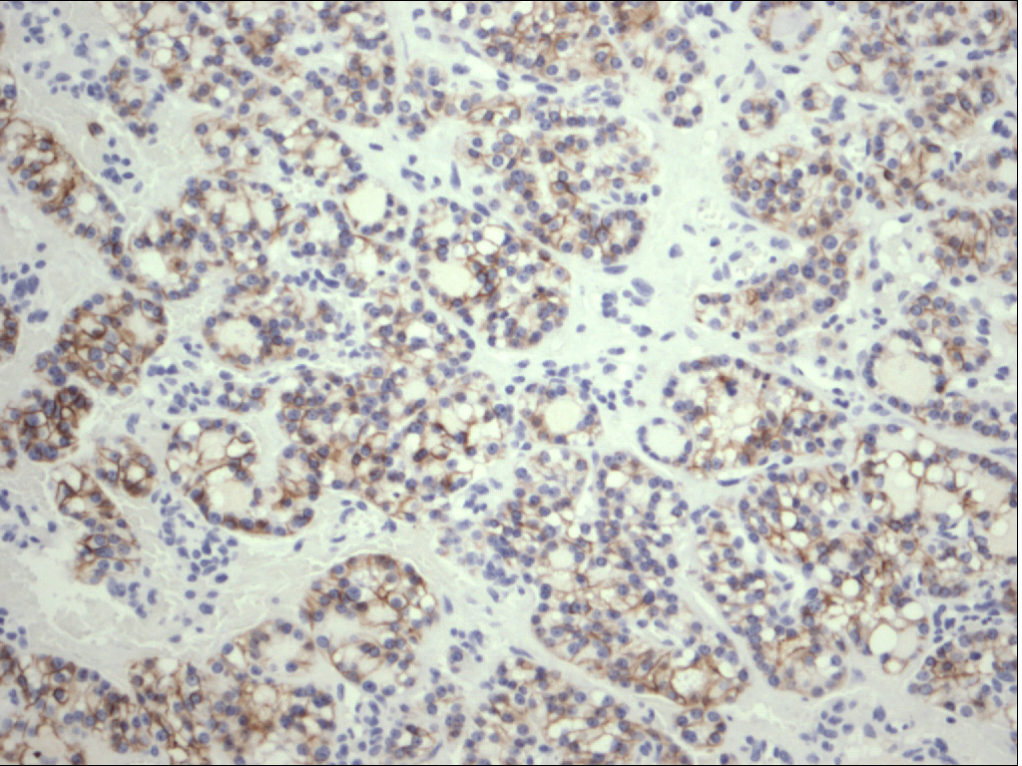
Follicular adenoma CD56.

**Figure 11:**
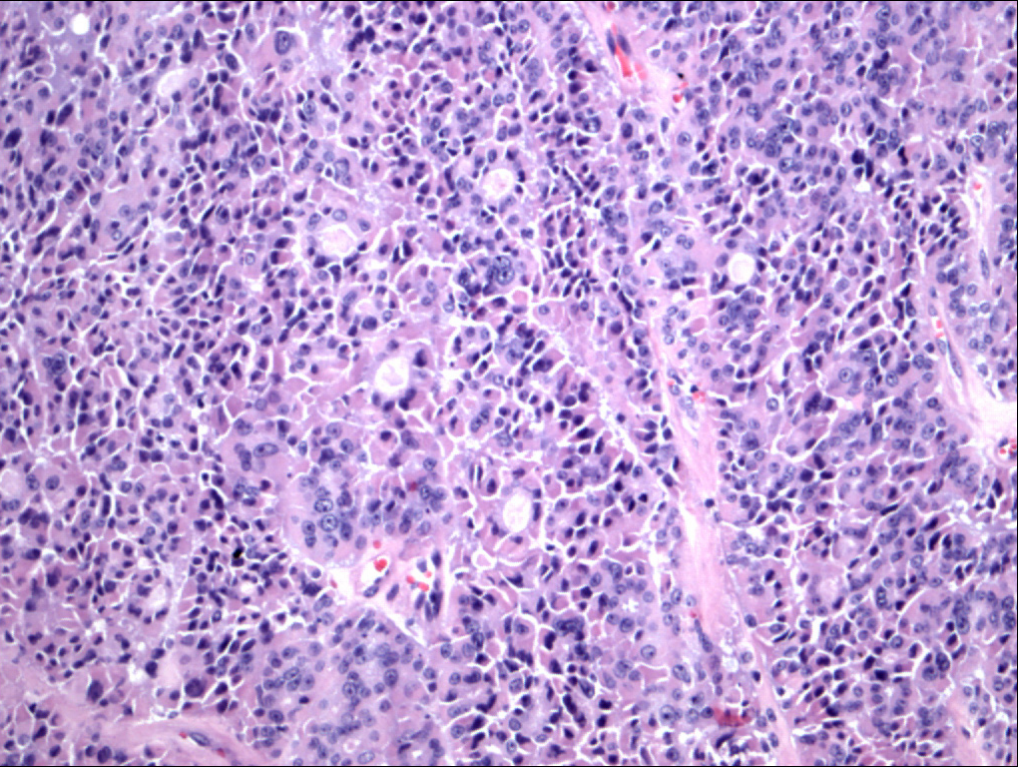
Hurthle cell carcinoma H&E.

**Figure 12:**
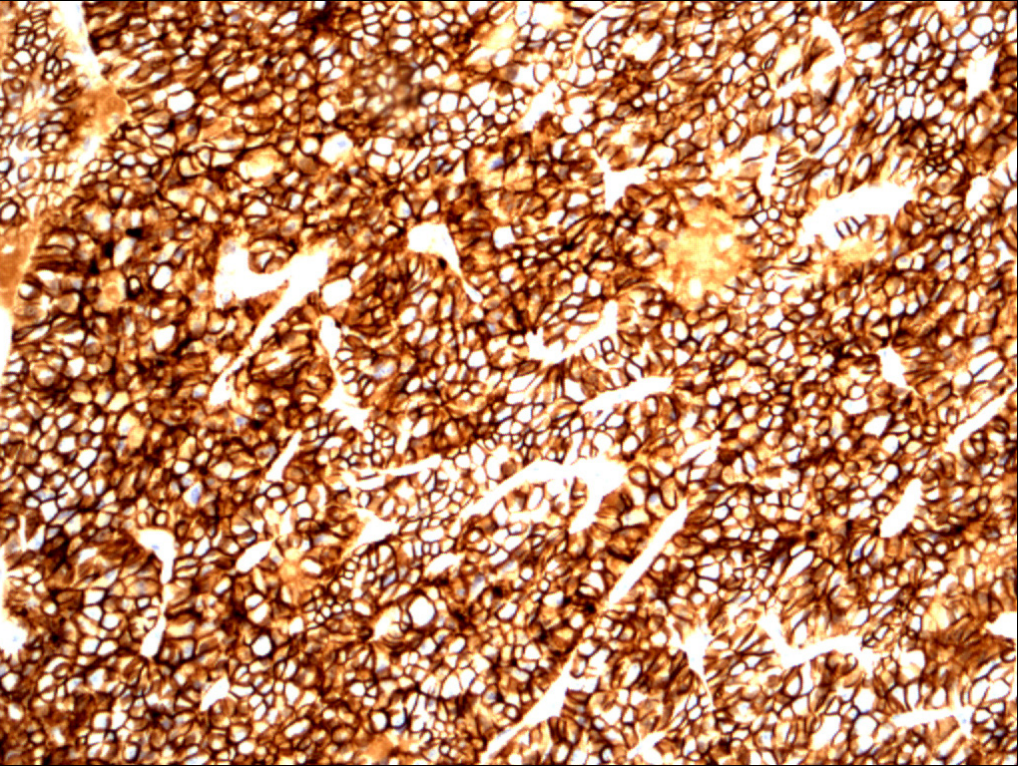
Hurthle cell carcinoma CD56.

Within individual benign group/normal thyrocytes and non-PTC lesions/tumors, CD56 expression showed consistently strong diffuse expression with no difference between different subgroups, including hyperplasia, thyroiditis (Figure 7, Figure 8), adenomas (Figure 9, Figure 10) and non PTC carcinomas (Figure 11, Figure 12). In all cases of the non-PTC group, the CD56 expression was membranous, crisp and diffuses involving 100% of the thyrocytes, in the lesion.

Within the PTC group, occasional CD56 positive cells were identified. In all cases, these cells were located at the tumor non-tumor interface i.e. at the periphery of the PTC. In all the PTC cases the tumors centers were completely devoid of any CD56 staining (0%). For all the cases of PTC, CD56 expression in individual cells did not exceed 5% of cells all at the periphery. To us these cells perhaps may represent the infiltrative tumor nontumor interface and may merely be residual non-tumor cells. We pointed out a <10% cut of for recording negative CD56 expression, to avoid the future dilemma of lack of quantization. It worth mentioning that, this occasional CD56 positive cells located at the tumor periphery (interface) were present in 6/15 of the classic, 20/23 of the follicular, 15/27 of the micro-papillary and of the 2/2 tall PTC variants. In the remaining PTC variants included in the study, CD56 expression was absent in all individual cells, centrally and peripherally (0%).

**Figure 13:**
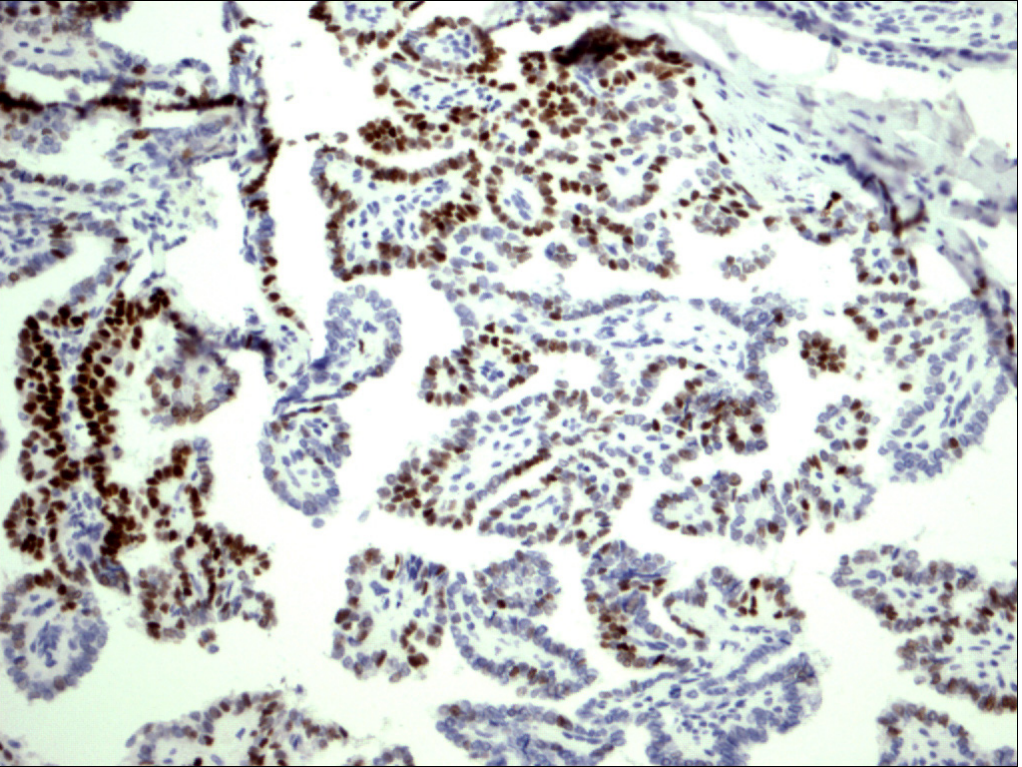
Classic papillary carcinoma P63.

**Figure 14:**
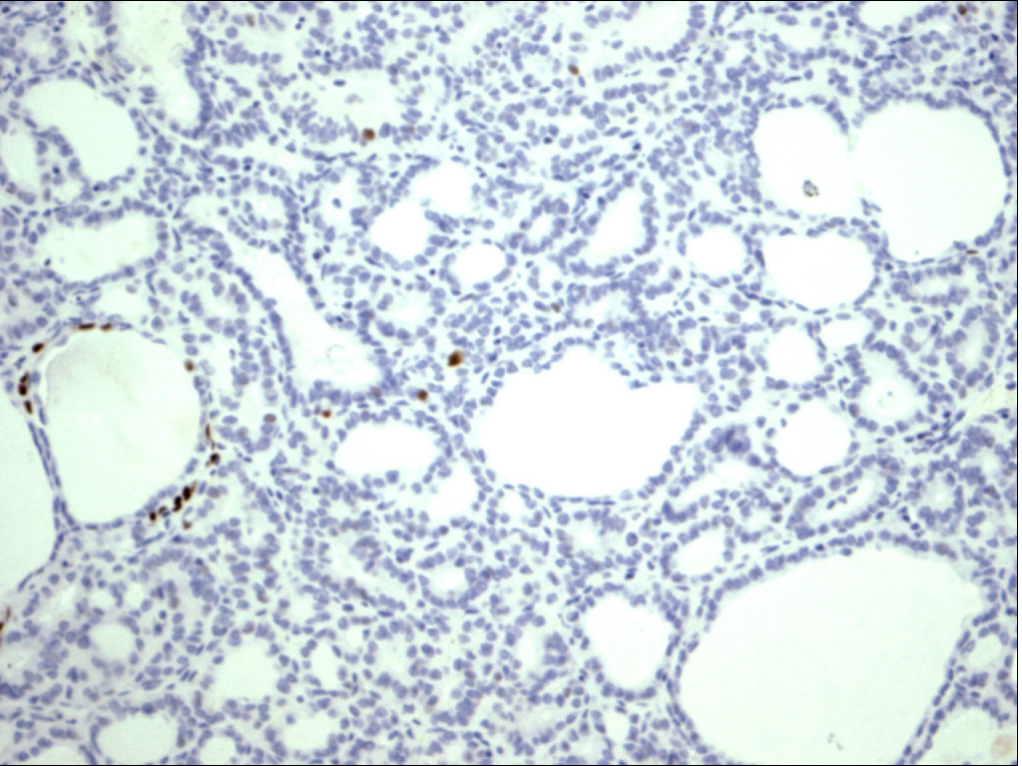
Follicular variant papillary carcinoma P63.

**Figure 15:**
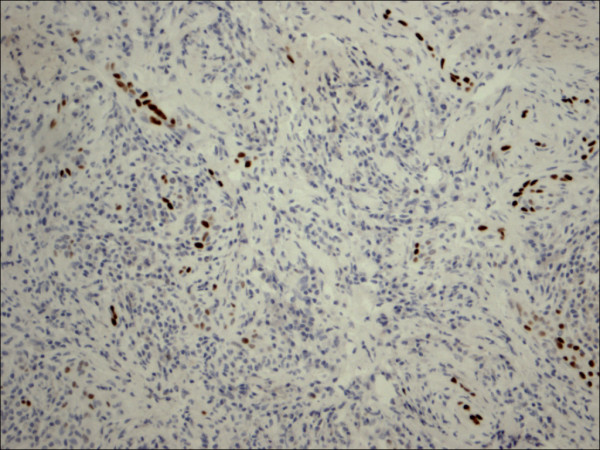
Papillary carcinoma in the setting of Hashimoto thyroiditis P63.

**Figure 16:**
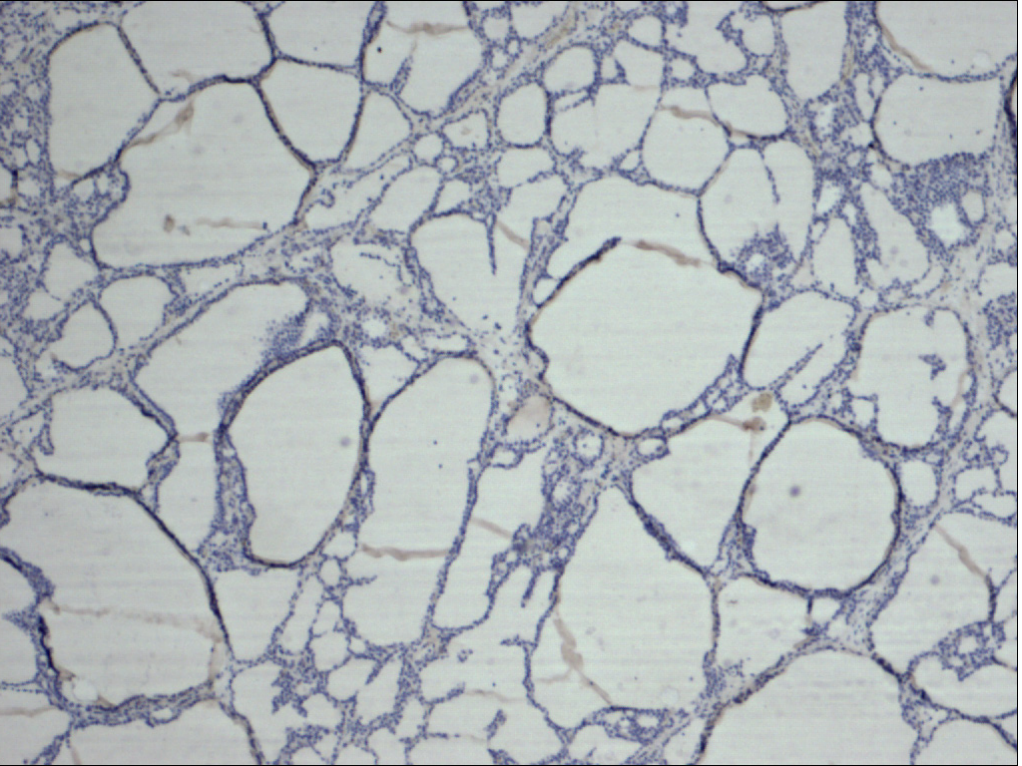
Thyrotoxicosis P63.

**Figure 17:**
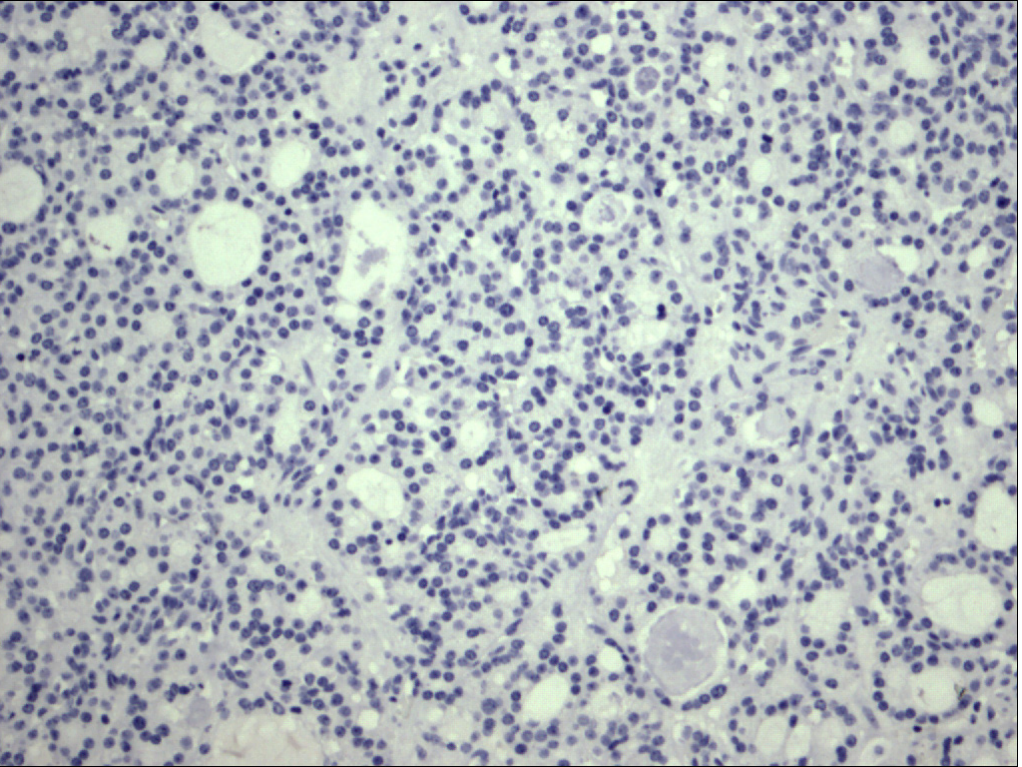
Follicular adenoma P63.

**Figure 18:**
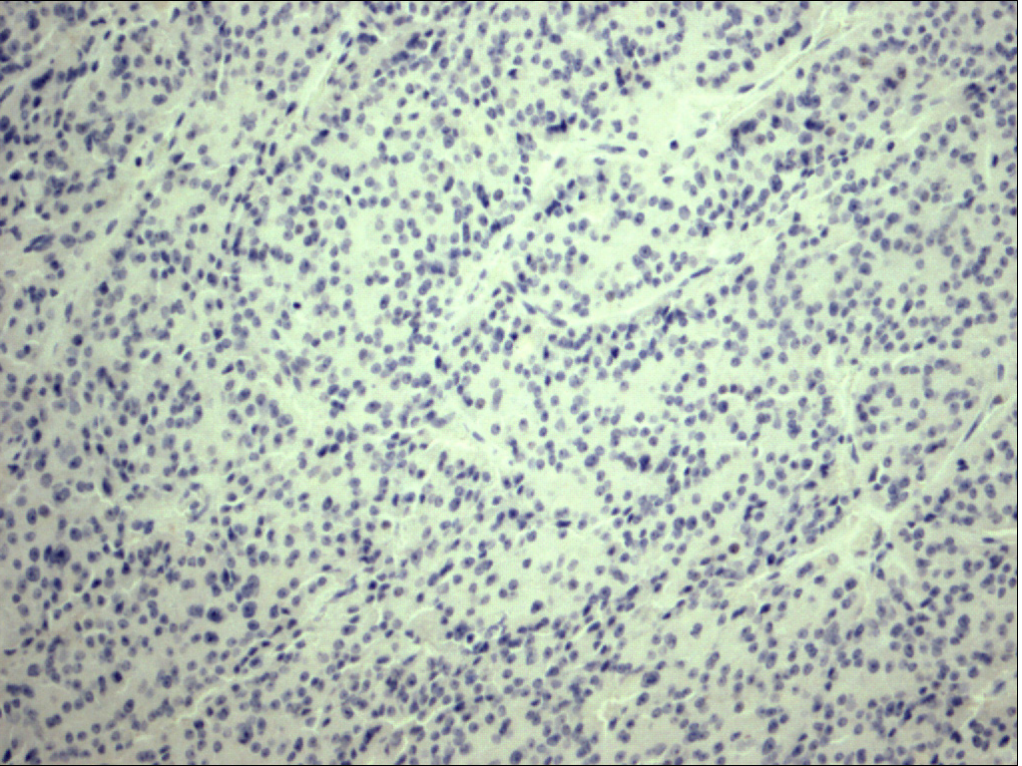
Hurthle cell carcinoma P63.

P63 showed selective focal positivity in PTC cases (Figure 13, Figure 14, Figure 15), in contrast to other non-PTC lesions/tumors (Figure 16, Figures 17, Figure 18). P63 expression was in 70% of cases of PTC and was consistently absent in all the non-PTC cases.

**Figure 19:**
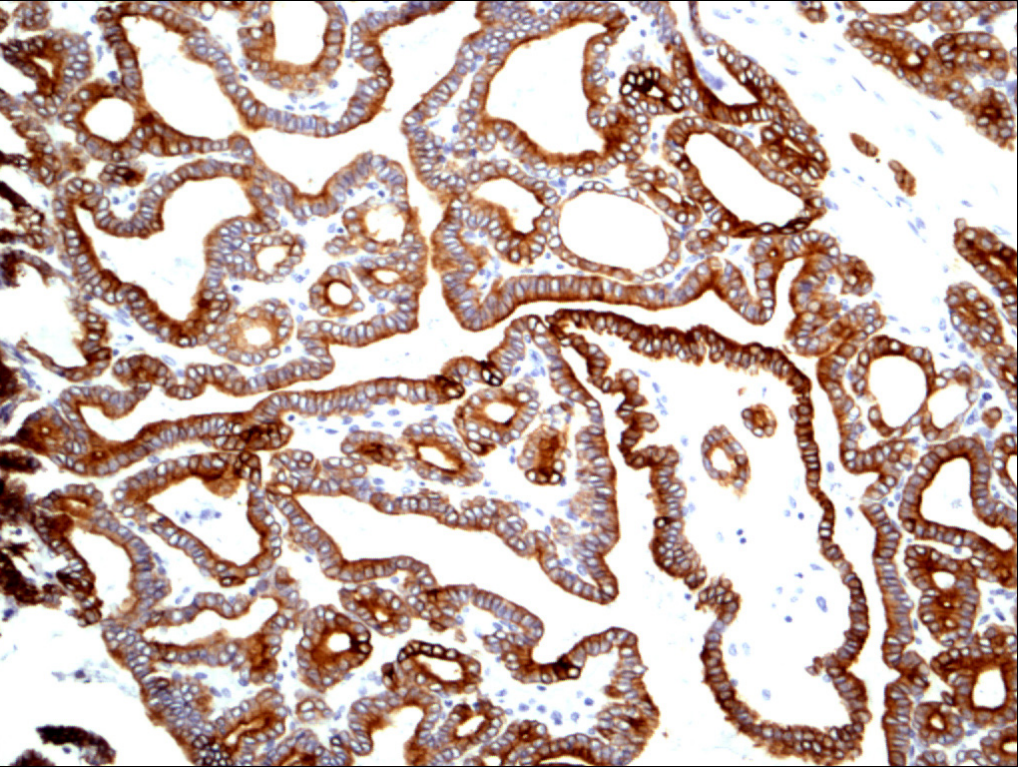
Classic papillary carcinoma CK19.

**Figure 20:**
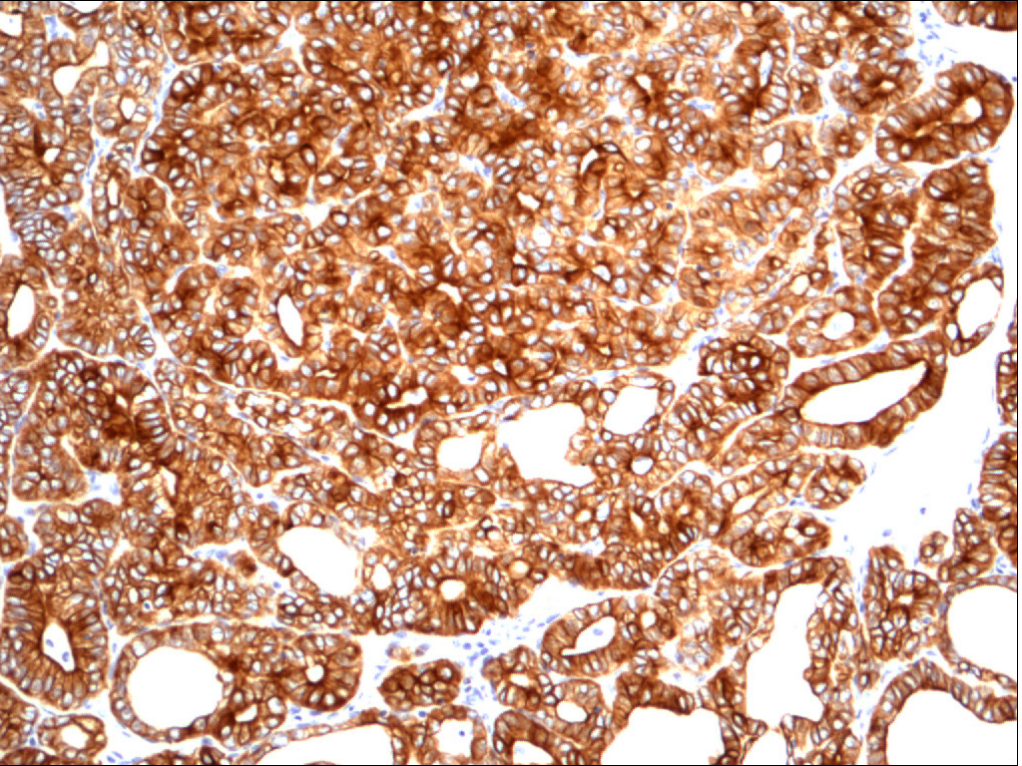
Follicular variant papillary carcinoma CK19.

**Figure 21:**
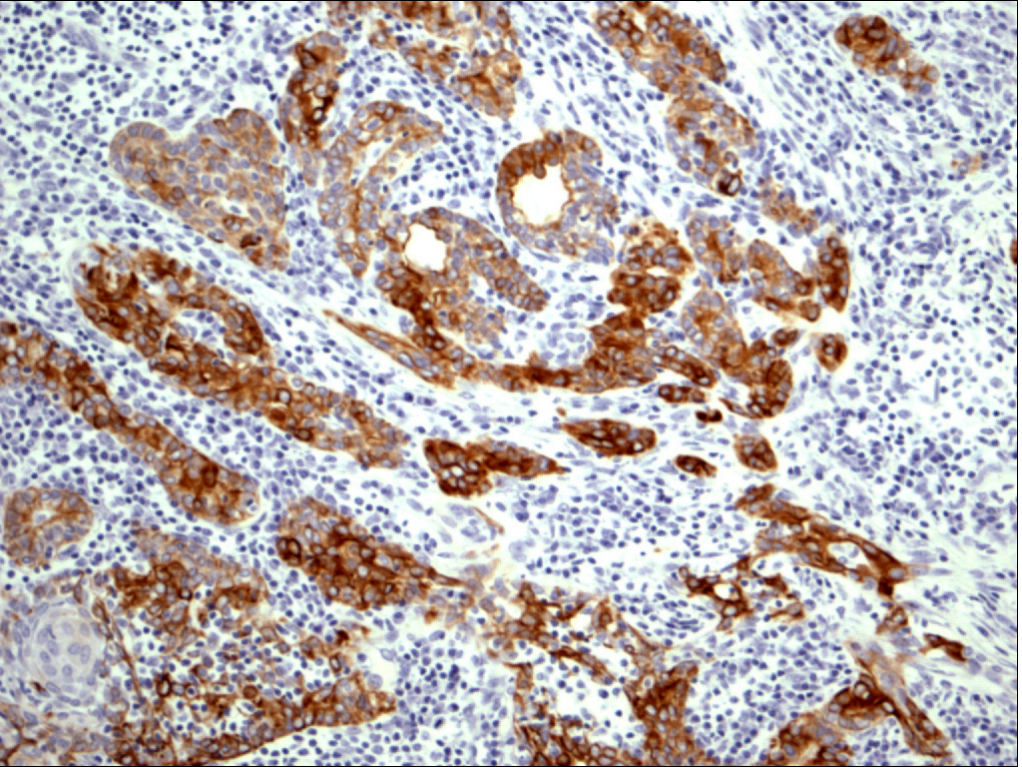
Papillary carcinoma in the setting of Hashimoto thyroiditis CK19.

**Figure 22:**
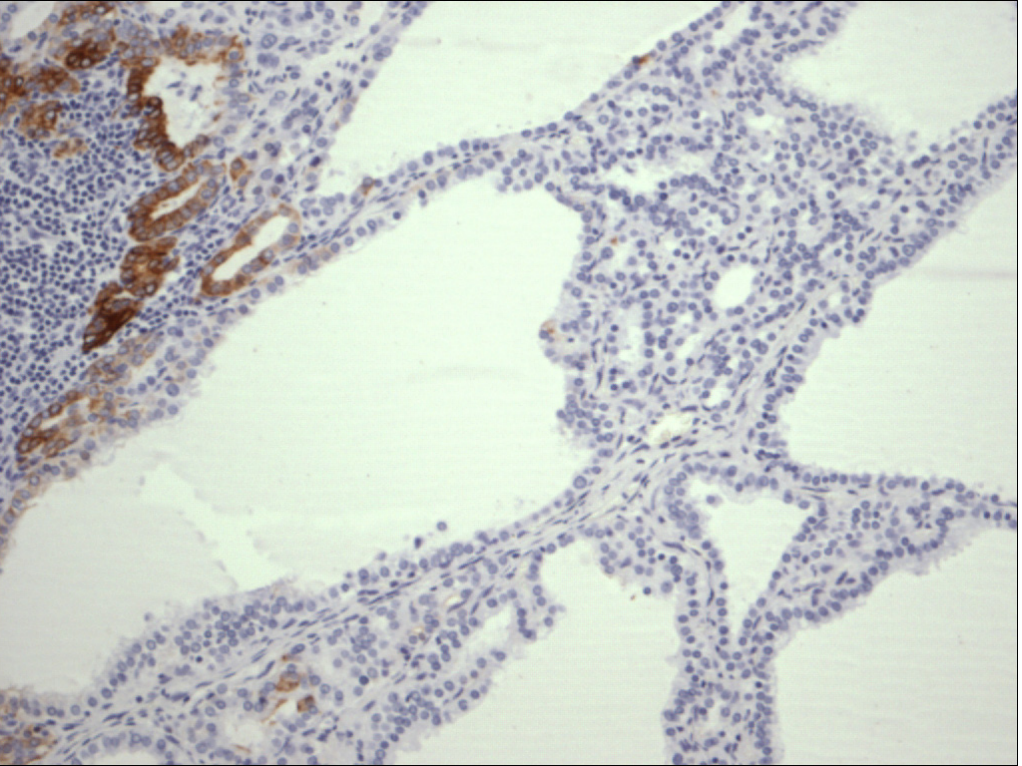
Thyrotoxicosis CK19.

**Figure 23:**
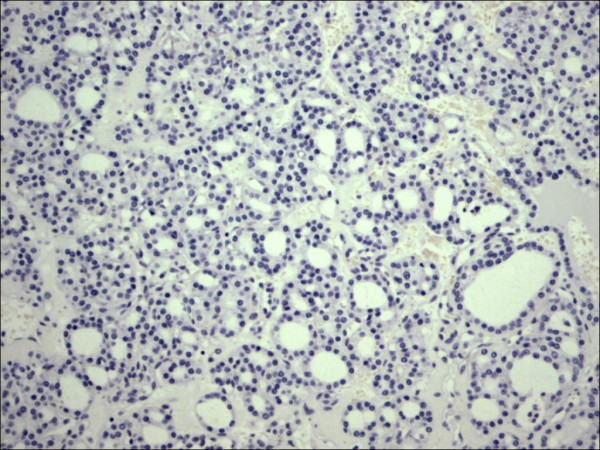
Follicular adenoma CK19.

**Figure 24:**
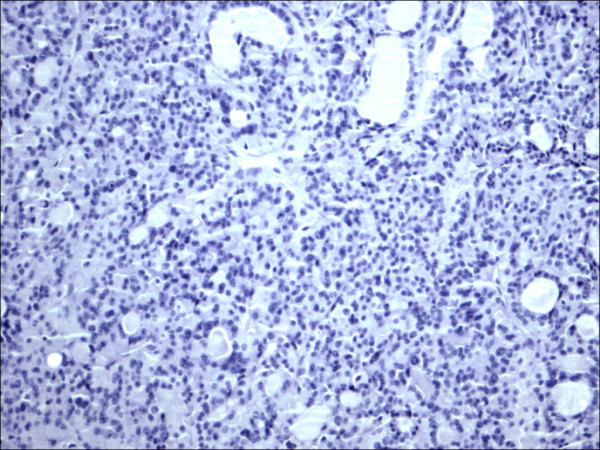
Hurthle cell carcinoma CK19.

CK 19 showed positive expression in PTC (Figure 19, Figure 20, Figure 21) and absent expression in non PTC lesions (Figure 22, Figure 23, Figure 24). However, CK19 expression accounted for 85% of cases of PTC and in 26% of cases of non-PTC lesions/tumors.

**Figure 25:**
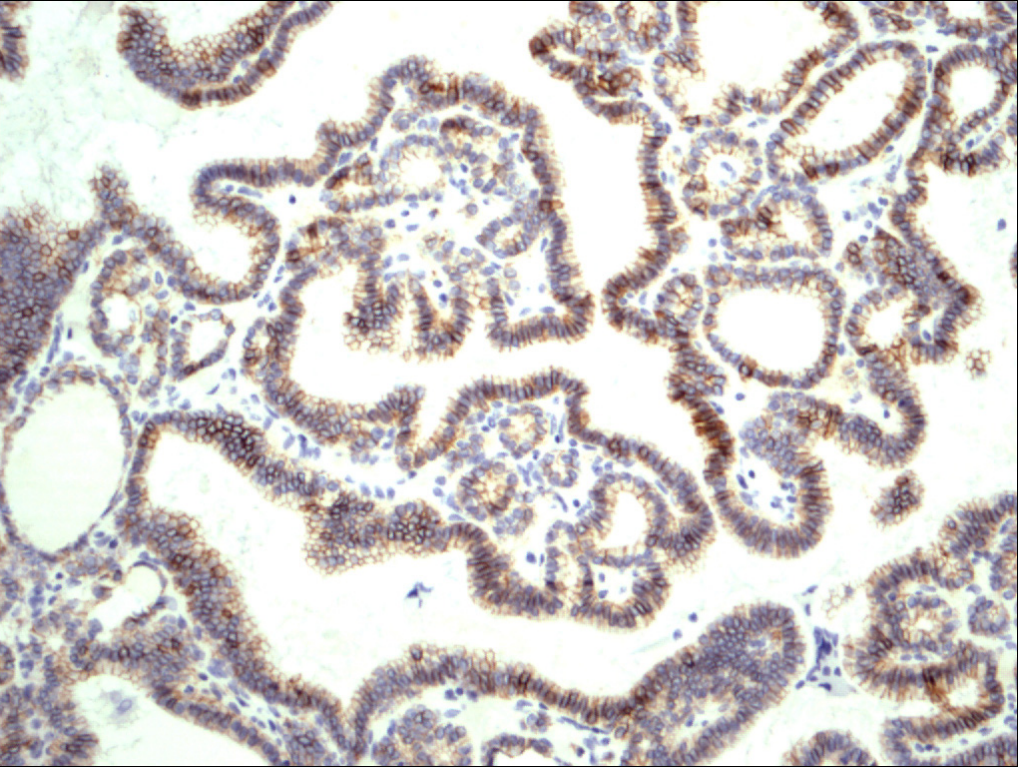
Classic papillary carcinoma E-Cadherin.

**Figure 26:**
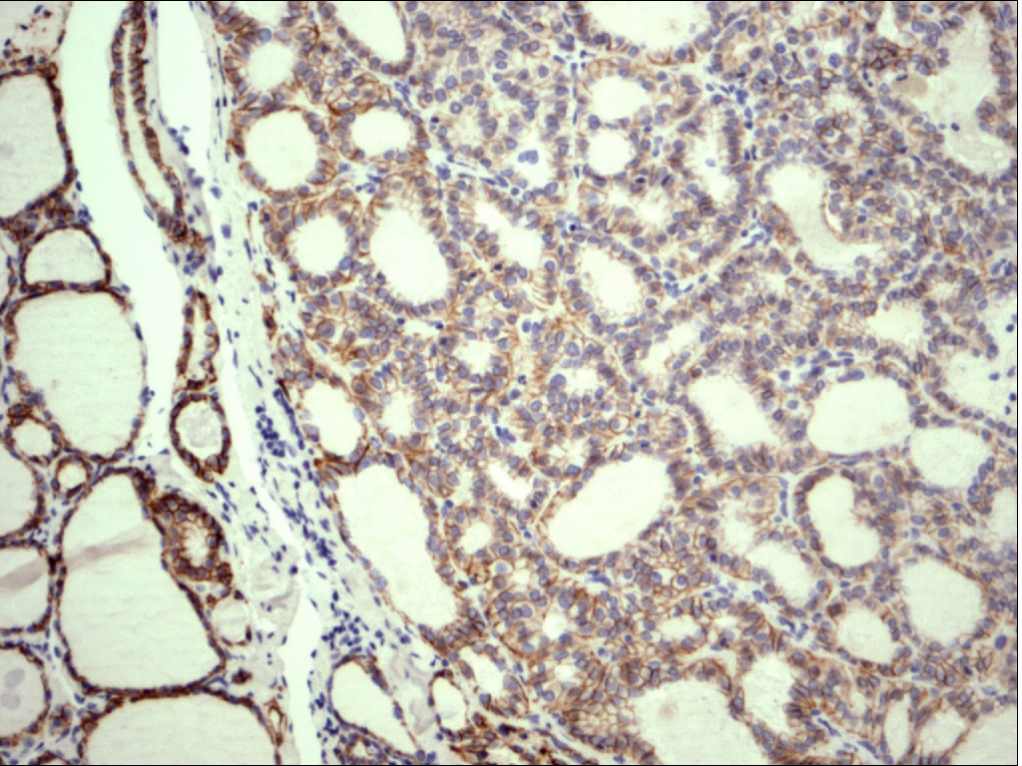
Follicular variant papillary carcinoma E-Cadherin.

**Figure 27:**
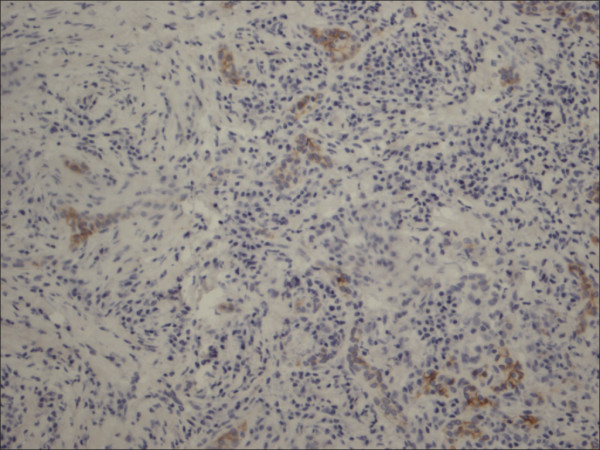
Papillary carcinoma in the setting of Hashimoto thyroiditis E-Cadherin.

**Figure 28:**
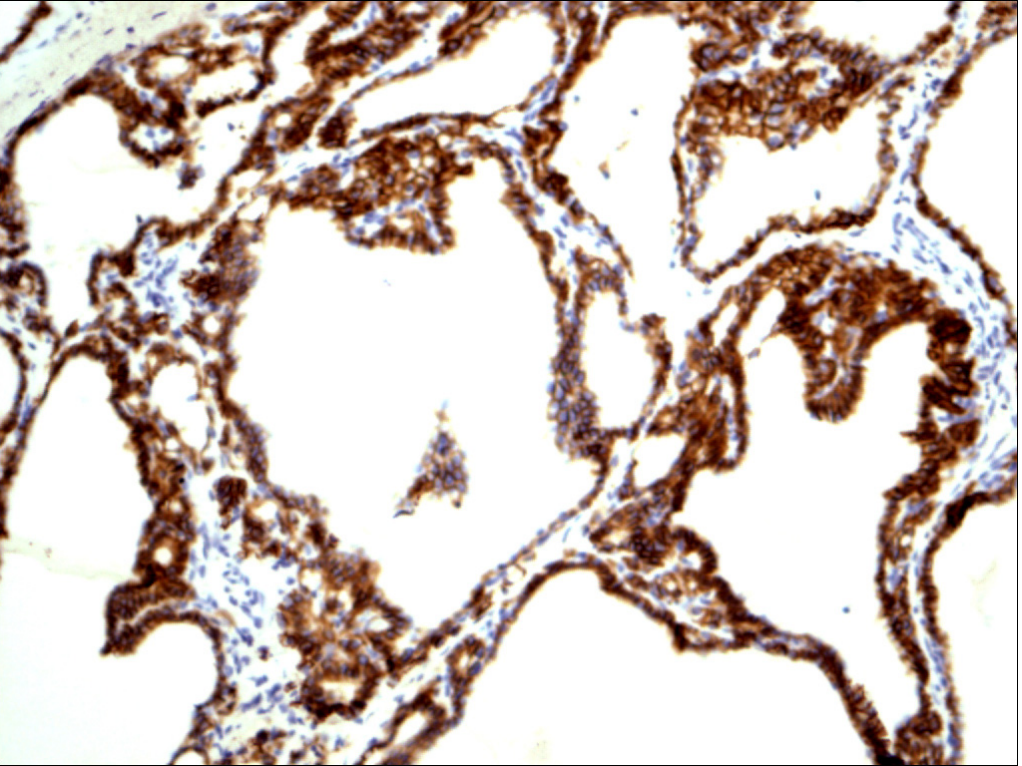
Thyrotoxicosis E-Cadherin.

**Figure 29:**
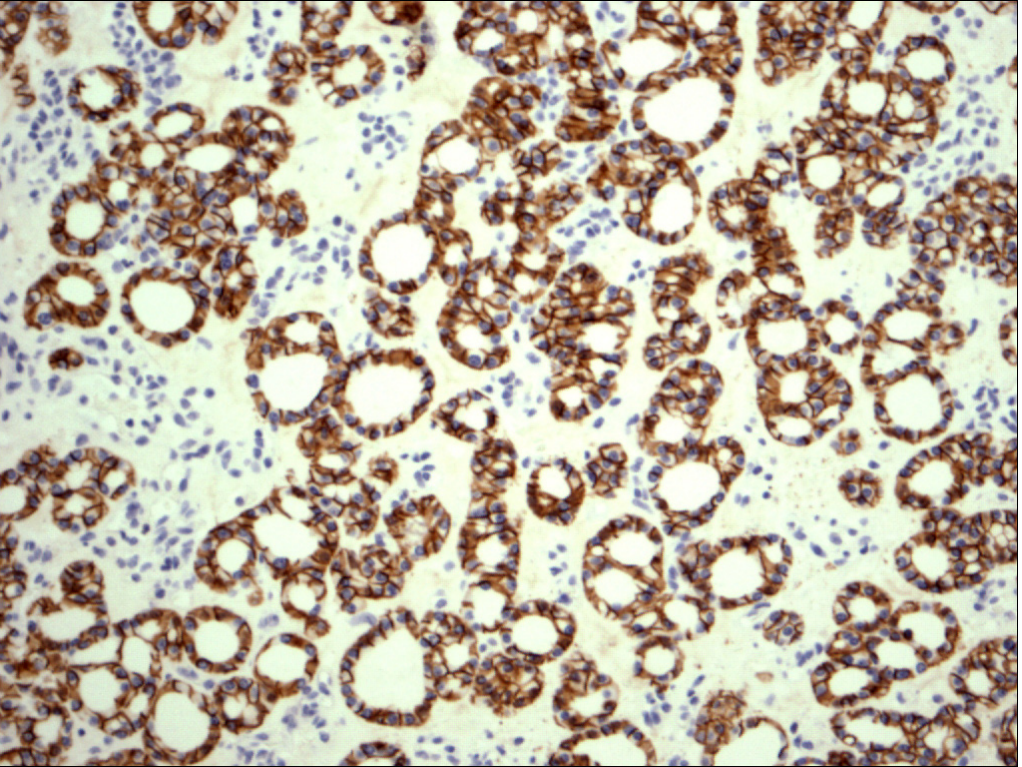
Follicular adenoma E-Cadherin.

**Figure 30:**
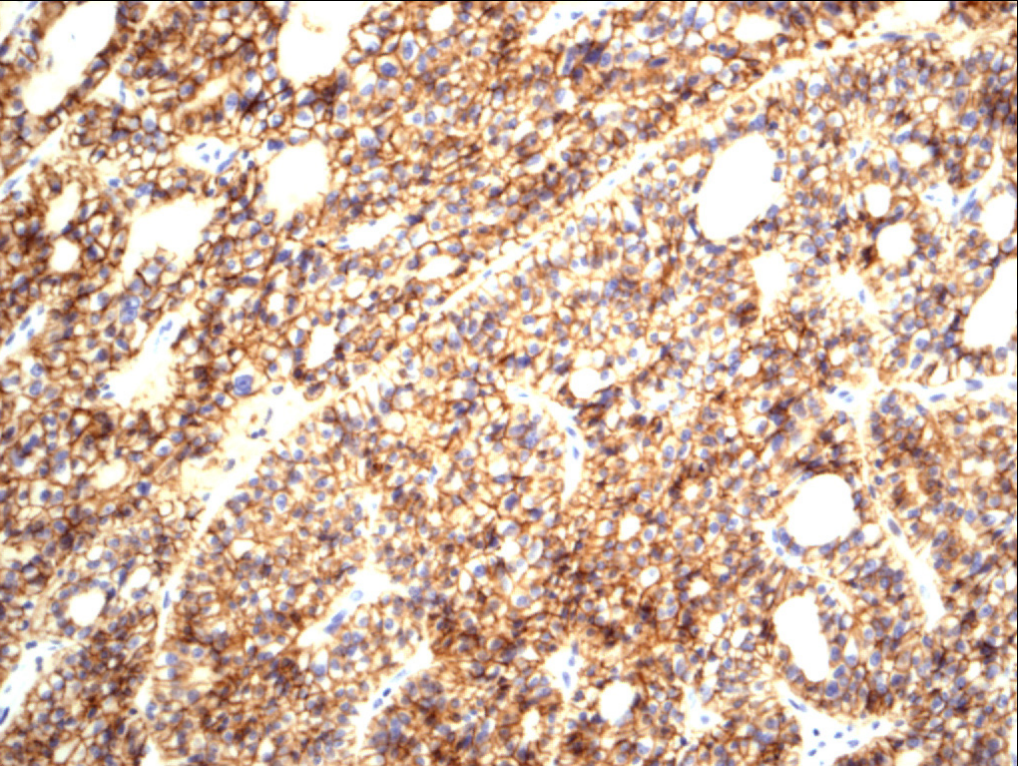
Hurthle cell carcinoma E-Cadherin.

E-cadherin showed consistent non-discriminatory expression in all cases included in the study (Figure 25, Figure 26, Figure 27, Figure 28, Figure 29, and Figure 30).

## Discussion

CD56 is a homophilic binding glycoprotein of the Ig-superfamily in which its antibody targets an isoform of the neural cell adhesion molecule (NCAM) and is expressed normally in NK cells, activated T cells, large granular lymphocytes, specific endocrine, and brain tissue [17,18]. The protein itself is believed to regulate cell motility, homophilic binding between neurons, stimulation of neurite outgrowth, in addition, fasciculation [19]. Since cell migration only takes place during development, wound healing, and tumor invasion, it should be noted that previous studies have shown that the expression of the gene reduces specific tumor invasion [20-22]. CD56 is present on follicular epithelial cells of the normal thyroid [23,24].

One of the most frequent difficulties in thyroid pathology is differentiating follicular variant of PTC from follicular adenoma. The inter observable variability in this area is well recognized. The differentiation is critical for the treatment and long-term management of the tumors. Despite the universal recognition of this variant, it is clearly lacking the minimal histological definition of the follicular variant of PTC. Instead of setting an objective minimal histological definition, in terms of field size or number of nuclei with PTC features, the literature resorts to an atypical category and introduces the term "well-differentiated tumor of uncertain malignant potential [25]." This term encompasses encapsulated tumors that have only "minor nuclear changes of the type seen in typical PTC. The problem with this term is that it really did not solve the inter observable variability between pathologists concerning the diagnosis of the follicular variant of PTC. Instead, it allowed safe expression of differences in opinion but certainly increased the chances of over-call and over-treatment of the follicular variant of PTC. Unfortunately, the immunoprofile of follicular derived lesions and neoplasms show some overlap and to the best of our knowledge, no single marker or even panel is 100% sensitive and 100% specific for PTC. Hence, the use of immunohistochemistry in the diagnosis of the follicular variant of PTC has to be used with extreme caution. A ecent study has investigated the expression of NCAM (CD56) in tissue sections of 61 cases of papillary carcinoma and in 14 lymph node metastases using immunohistochemistry [26]. In this study, low or absent expression of CD56 was noted in PTC using immunohistochemistry and PCR [26]. In the current study, CD56 was extremely useful in the distinction between PTC, including the follicular variant and other follicular lesions/neoplasms, as well as PTC mimickers' such as Hashimoto and lymphocytic thyroiditis and follicular adenoma.

We point out that we excluded those cases (4 cases), that we disagreed upon and those were diagnosed as well-differentiated follicular neoplasm of uncertain malignant potential. The rational behind their exclusion is that those cases are rare and highly controversial, and perhaps a large series of such cases should be evaluated by a group of experts in regards to the diagnosis and applicability of CD56, P63, E-cadherin and CK19. Our study shows that within the thyroid gland, the use of an immunohistochemical panel formed of CD56, E-cadherin, and P63 is extremely helpful in selecting cases of PTC from other follicular cell-derived thyroid lesions/tumors, with 100% sensitivity and 100% specificity. However, this is certainly not applicable outside of the thyroid gland, i.e. within metastatic sites.

Hence, we suggest that immunohistochemistry using this panel is helpful in the diagnosis of PTC including the follicular variant of PTC. E-Cadherin immunhistochemistry is noncontributory.

## Conclusion

Lack of CD56 expression in follicular cell-derived PTC is consistent, specific, and extremely sensitive. P63 is specific but less sensitive for diagnosing PTC. CK19 is lessspecific than both of the previously mentioned markers are, however it is more sensitive than P63 but less sensitive than CD56. E-cadherin is of no value in the diagnosis of PTC and differentiating it from other follicular lesions/neoplasm.

Using an immunohistochemical panel formed of CD56, P63 and CK19 in the diagnosis of PTC, particularly follicular variant, is extremely useful. Evaluation of CD56 and P63 immunohistochemical expression by a group of expert thyroid pathologists, on a larger series, especially those included in the categories of follicular neoplasm of uncertain malignant potential and follicular carcinomas, may provide a more objective diagnosis of PTC.

## Authors' contributions

DD has written the manuscript, reviewed the clinical and pathological data of the cases, and interpreted the immunohistochemical results. AN has reviewed the manuscript, particularly as regards the clinical context, and analysis of the clinical results. SA has reviewed the pathological data of the cases, interpreted the immunohistochemical results, and revised the manuscript. All authors read and approved the final manuscript.
